# Postoperative “Chimney” for Unintentional Renal Artery Occlusion after EVAR

**DOI:** 10.1155/2014/170198

**Published:** 2014-11-16

**Authors:** Marco Franchin, Federico Fontana, Filippo Piacentino, Matteo Tozzi, Gabriele Piffaretti

**Affiliations:** ^1^Vascular Surgery, Department of Surgery and Morphological Sciences, Circolo University Hospital, University of Insubria School of Medicine, Via Guicciardini 9, 21100 Varese, Italy; ^2^Interventional Radiology, Department of Surgery and Morphological Sciences, Circolo University Hospital, University of Insubria School of Medicine, Via Guicciardini 9, 21100 Varese, Italy

## Abstract

Renal artery obstruction during endovascular repair of abdominal aortic aneurysm using standard device is a rare but life-threatening complication and should be recognized and repaired rapidly in order to maintain renal function. Both conventional surgery and endovascular stenting have been reported. We report a case of late postoperative bilateral “chimney” to resolve a bilateral thrombosis of the renal artery following an uncomplicated endovascular aortic repair.

## 1. Introduction

Acute kidney injury (AKI) remains a known complication after endovascular abdominal aortic aneurysm repair (EVAR): multiple factors may be involved in postoperative AKI including contrast-induced nephropathy, atheroembolism, occlusion of accessory renal arteries, or unintentional overstenting of the renal arteries [1, 2].

Generally, renal artery occlusion due to endograft (EG) overstenting is recognized rapidly and could be treated immediately during the procedure; in contrast, unexpected renal artery occlusion may lead to prolonged ischemic damage and potential permanent injury requiring hemodialysis [3, 4].

In this report, we describe the occurrence of an unintentional and unexpected bilateral renal artery overstenting successfully managed with a bilateral renal artery “chimney” stenting in the late postoperative course.

## 2. Case Report

**Figure 1:**
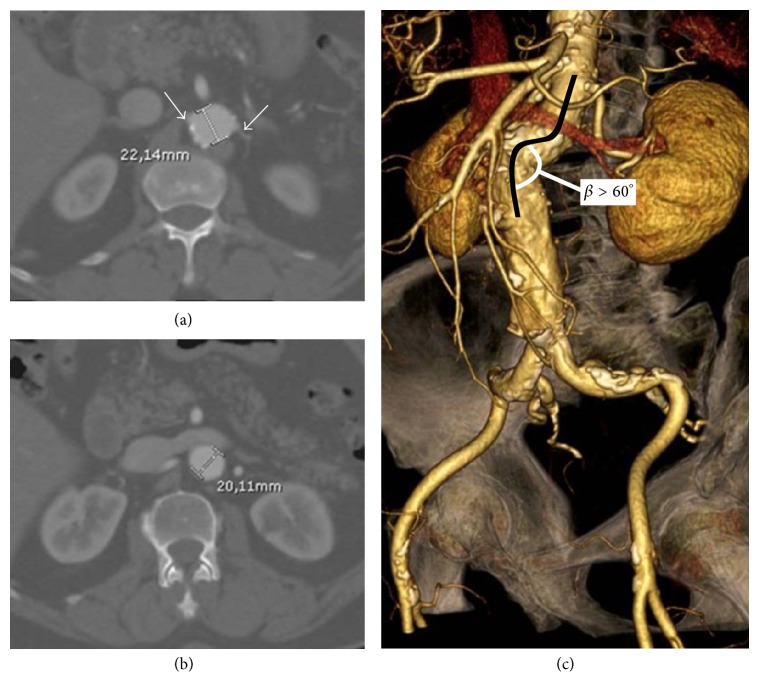
Preliminary CT-A showing (a, b) the reversed tapered shape of the proximal neck (arrows: renal arteries origin) and the severe *β*-angle (c).

**Figure 2:**
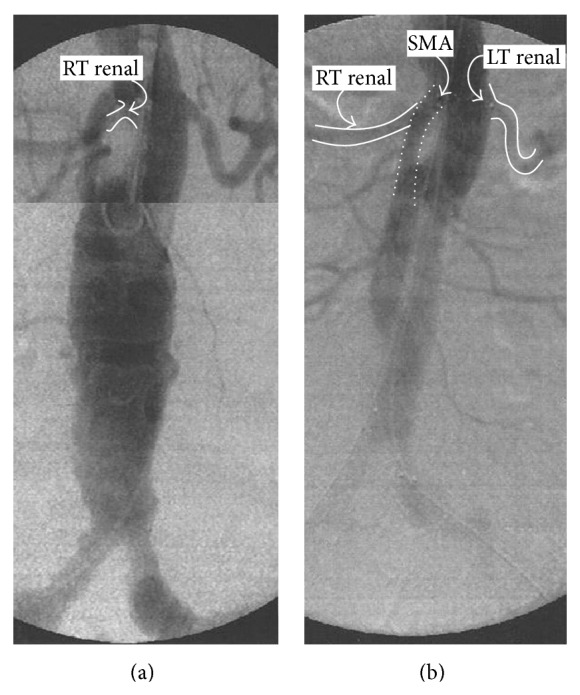
Predeployment preliminary angiography (a). Final control (b) showed the patency of both the renal arteries (arrows).

**Figure 3:**
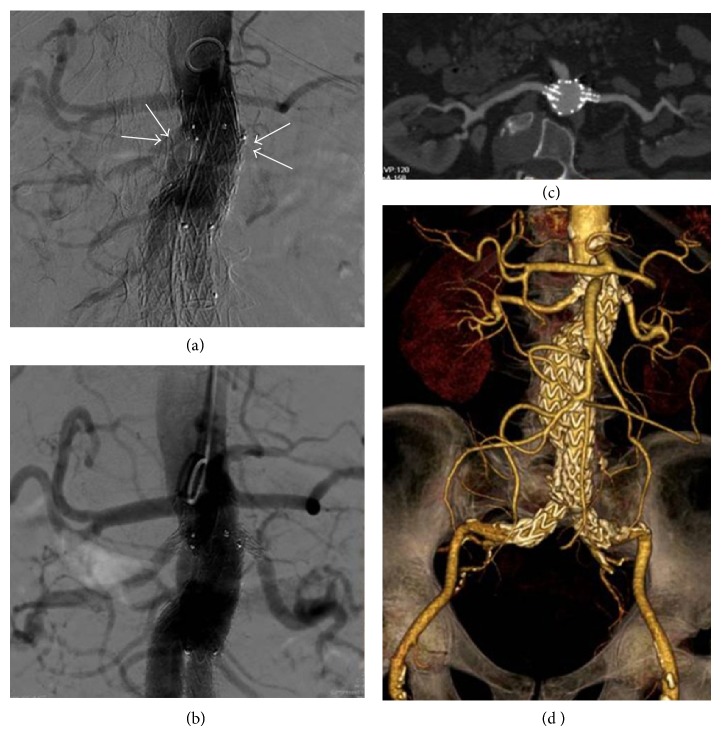
Selective angiography at the proximal extremity of the EG (a) showed the overstenting (arrows) of the origin of both the renal arteries. Complete revascularization after bilateral stenting (b). Follow-up CT-A: complete reperfusion of the parenchyma (c) as well as persistent exclusion of the aneurysm and absence of endoleak (d).

A 73-year-old man was admitted to our department with a diagnosis of an asymptomatic 57 mm fusiform abdominal aortic aneurysm (AAA). Medical history was notable for obesity, hypertension, chronic depressed (<30%) left heart dysfunction, and chronic obstructive pulmonary disease. Preoperative computed tomography angiography (CT-A) highlighted the presence of a hostile proximal aortic neck characterized by a reversed tapered shape (Figures 1(a) and 1(b)) plus an acute *β*-angle > 60° (Figure 1(c)). In the operating room, a 28 mm transrenal bifurcated EG (Endurant-Medtronic; Santa Rosa-CA; USA) was implanted through conventional bilateral groin cut-down. Final angiography confirmed the complete exclusion of the AAA with no evidence of proximal or distal endoleak, as well as the visualization of the renal arteries (Figures 2(a) and 2(b)). The immediate postoperative serum creatinine level was 1.48 mg/dL (range, 0.6–1.3 mg/dL; preoperative level: 1.36 mg/dL). Twelve hours later, a progressive reduction of urine output was noted; at that time, serum creatinine level increased to 3.26 mg/dL, configuring a grade 4 AKI according to the Aneurysm Renal Injury Score (ARISe) [5]. Angiography was performed immediately thereafter: it showed the occlusion of the renal artery, bilaterally. During the same procedure, we attempted an endovascular revascularization of the renal arteries, which failed because of the acute onset of a high-response atrial fibrillation causing hemodynamic instability and acute respiratory distress. The patient was transferred in the intensive care unit and started a temporary renal replacement therapy and amiodarone (Cordarone-Sanofi-Aventis; Milano-Italy) intravenously. Four days later, hemodynamic stability was recovered; at that time, a percutaneous left transbrachial approach was used to catheterize selectively the renal arteries (Figure 3(a)) with a 0.014′′ guidewire (Stabilizer-Cordis, Miami Lakes, FL, USA) coupled with a 4F vertebral catheter (Cordis; Miami Lakes-FL; USA). A 5 × 15 mm bare metal stent (Genesis*-*Cordis; Miami Lakes-FL; USA) was used bilaterally with the complete restoration of renal flow into the parenchyma (Figure 3(b)). The subsequent postoperative course was uneventful: the urine output improved progressively. He was discharged on day 6 postoperatively on acetylsalicylic acid (Cardioaspirin-Bayer; Milano-Italy) 100 mg/die* ad infinitum*. He was last seen eighteen months later; the patient is still alive, asymptomatic, and the follow-up CT-A confirmed the complete exclusion of the aneurysm without endoleaks and the patency of the renal stents with preservation of visceral flow (Figures 3(c) and 3(d)). At that time serum creatinine level was 1.38 mg/dL.

## 3. Discussion

The starting points for discussion of our case are either technical or clinical: they concern the underhanded pathogenesis of the renal artery occlusion, the feasibility of a postoperative “chimney” technique to overcome the renal artery ostia overstenting, and the recovery of the renal function 96 hours after the onset of the acute kidney injury.

As all the innovative techniques, even EVAR has brought new complications such as endoleaks and migration; some others are shared with conventional aortic repair such as renal artery occlusion [6]. Acute kidney injury is a serious complication and harbinger of poor prognosis after EVAR [7, 8]: despite renal artery occlusion during EVAR remaining an uncommon complication, as EVAR gains popularity, the incidence of EVAR-associated renal artery obstruction may increase.

EVAR-related renal artery occlusion is generally found intraoperatively following an EG maldeployment; however, it has been suggested that also those occlusions detected during the follow-up could be considered occluded since the initial operation [9–11]. Inan et al. [12] described a case of intraoperative bilateral renal artery occlusion: the cause was a proximal migration of a bifurcated EG, an event that is anecdotal and mainly due to an iatrogenic upward thrust of the device during contralateralcannulation. When suprarenal stents were first introduced, there was concern that they would induce hyperplasia and narrow the renal orifices; up to date, insufficient high-level evidence has decreed for or against proximal transrenal fixation on EVAR-related renal occlusion [13–15]. In our case, as probably occurs in most of the cases, the mechanism that caused the renal artery thrombosis was an underhanded partial obstruction that can be difficult to identify on intraoperative angiograms. We had an uncomplicated deployment with visualization of the renal arteries at completion angiography: the reason that may explain the delayed onset of the thrombosis of the renal arteries can be ascribed to the imperceptible filling defect in the renal artery profile even when the EG covers much of the orifice so that the renal artery lumen often fills with contrast-enhanced blood. Furthermore, the partial obstructions of renal arteries by the graft material or the struts of the suprarenal hooks were potentially masked by intraoperative anticoagulation.

Currently, there is no consensus about the treatment strategy of operative treatment and outcomes after prolonged renal artery occlusion. We believe that an attempt to revascularize the overstented renal arteries should have been mandatory in our case. Twine and Boyle [5] reported the occlusion of both renal arteries in the early postoperative period of an ordinary infrarenal EVAR resulting in dialysis-dependent kidney injury. Our case shows that urgent revascularization could be effective for this type of complication. Maybe the favorable outcome was facilitated by the early development of a rich collateral circulation that has been studied to come up most commonly from the periureteral, peripelvic, and adrenal vessels and that can maintain viability of nephrons at sub-filtration arterial pressures; furthermore, it provides the rationale for rescue intervention after total renal artery occlusion, even when the diagnosis has been delayed [16, 17].

Both open and endovascular techniques may be used as procedures to treat this uncommon, but important, especially if we consider the rapid and steady increase of EVARs either to treat conventional anatomies or complex aortic necks [18]. Open conversion still remains an option: when they covered the renal arteries for a number of weeks Adu et al. [18] proposed a flowchart with specific vascular bypasses depending on the patency status of the celiac trunk. In contrast, endovascular revascularization is primarily determined by accessibility of the renal orifices [4, 10, 12]. Similar to our case, Hedayati et al. [11] reported two cases of renal artery occlusion treated one week after a transrenal EVAR with renal artery stenting using a transfemoral approach which led to symptom resolution and recovery of renal function. In the present case the femoral approach has not been successful; in contrast, the second attempt using a brachial approach has led to an easier and more rapid catheterization of both renal arteries, perhaps more so for the presence of the transrenal bare fixation and despite the greater length of the shaft affecting its maneuverability.

## 4. Conclusion

Lessons learned from our case are never settled on the contrast effect into renal arteries at the time of completion angiography, and revascularization is still an option even when EVAR-related occlusion of the renal arteries has a delayed onset. Postoperative “chimney” might be considered a viable alternative if the renal artery overstenting is not completely occlusive also because it does not preclude a subsequent conventional bypass attempt.
